# Patient Use, Experience, and Satisfaction With Telehealth in an Australian Population (Reimagining Health Care): Web-Based Survey Study

**DOI:** 10.2196/45016

**Published:** 2023-08-17

**Authors:** Elizabeth Thomas, Crystal Man Ying Lee, Richard Norman, Leanne Wells, Tim Shaw, Julia Nesbitt, Isobel Frean, Luke Baxby, Sabine Bennett, Suzanne Robinson

**Affiliations:** 1 School of Population Health Curtin University Bentley Australia; 2 Medical School The University of Western Australia Perth Australia; 3 Consumers Health Forum of Australia Canberra Australia; 4 Charles Perkins Centre University of Sydney Sydney Australia; 5 Digital Health Cooperative Research Centre Sydney Australia; 6 Deloitte Brisbane Australia; 7 Deloitte Sydney Australia; 8 Deakin Health Economics, Institute for Health Transformation School of Health and Social Development, Faculty of Health Deakin University Melbourne Australia

**Keywords:** telehealth, consumer experience, demographic influence, population survey, health care survey, telemedicine, Australia, participant recruitment

## Abstract

**Background:**

The COVID-19 pandemic triggered a rapid scale-up of telehealth services in Australia as a means to provide continued care through periods of physical restrictions. The factors that influence engagement in telehealth remain unclear.

**Objective:**

The purpose of this study is to understand the experience of Australian people who engaged in a telehealth consultation during the pandemic period (2020-2021) and the demographic factors that influence engagement.

**Methods:**

A web-based survey was distributed to Australians aged over 18 years that included 4 questions on frequency and type of clinical consultation, including with a general practitioner (GP), specialist, allied health, or nurse; 1 question on the experience of telehealth; and 2 questions on the quality of and satisfaction with telehealth. Statistical analysis included proportion of responses (of positive responses where a Likert scale was used) and regression analyses to determine the effect of demographic variables.

**Results:**

Of the 1820 participants who completed the survey, 88.3% (1607/1820) had engaged in a health care consultation of some type in the previous 12 months, and 69.3% (1114/1607) of those had used telehealth. The most common type of consultation was with a GP (959/1114, 86.1%). Older people were more likely to have had a health care consultation but less likely to have had a telehealth consultation. There was no difference in use of telehealth between metropolitan and nonmetropolitan regions; however, people with a bachelor’s degree or above were more likely to have used telehealth and to report a positive experience. A total of 87% (977/1114) of participants agreed or strongly agreed that they had received the information they required from their consultation, 71% (797/1114) agreed or strongly agreed that the outcome of their consultation was the same as it would have been face-to-face, 84% (931/1114) agreed or strongly agreed that the doctor or health care provider made them feel comfortable, 83% (924/1114) agreed or strongly agreed that the doctor or health care provider was equally as knowledgeable as providers they have seen in person; 57% (629/1114) of respondents reported that they would not have been able to access their health consultation if it were not for telehealth; 69% (765/1114) of respondents reported that they were satisfied with their telehealth consultation, and 60% (671/1114) reported that they would choose to continue to use telehealth in the future.

**Conclusions:**

There was a relatively high level of engagement with telehealth over the 12 months leading up to the study period, and the majority of participants reported a positive experience and satisfaction with their telehealth consultation. While there was no indication that remoteness influenced telehealth usage, there remains work to be done to improve access to older people and those with less than a bachelor’s degree.

## Introduction

Telehealth is a mode of remote delivery of health care that has increased in usefulness across primary, specialist, and allied health care in the last 15 years. The term *telehealth* is often used to refer to multiple modes of delivery, including telephone, video, and other digital forms of care such as monitoring tools connected to wearable devices [1]. The application of telehealth models of care is diverse, ranging from specialist consultations to regular primary care follow-ups for chronic conditions, such as cardiovascular disease or diabetes [2-5].

In the last 3 years, in response to the COVID-19 pandemic, there has been a rapid increase in the use of telehealth as a mode of primary and specialist health care globally [2,6-8]. In Australia, this has been fostered by the introduction of a public insurance (Medicare) item that enabled clinicians to quickly adapt to telehealth for regular consultations without causing their patients to incur an out-of-pocket cost [9]. While this has been necessary to enable care to continue through restrictions imposed by the pandemic, the impact of this on health outcomes and understanding patient perspectives on the growing spectrum of telehealth services available remains an area of investigation.

A 2020 report on Australian experiences with telehealth found that a majority of people have had a positive experience with telehealth and can perceive a place for telehealth beyond the COVID-19 pandemic [10]. The review by Eze and colleagues [11] supported this for all Organisation for Economic Co-operation and Development countries and showed that telehealth showed benefits for cost-effectiveness and patient outcomes for some conditions. However existing research has lacked detail on the types of consultations patients were undergoing with telehealth, such as primary or specialist care, and there is a need to further understand the demographic biases that are shown to limit engagement with telehealth in other populations [12].

The Reimagining Health Care survey involved a population-based questionnaire to almost 2000 Australian adults during the period of June to September 2021 [13]. The survey included 48 questions about individuals’ use, perception of, and satisfaction with telehealth across various clinical domains, including quality of care and technical quality, as well as demographic factors that influence engagement and satisfaction. The aim of this paper is to report on the outcomes of this research with respect to patient experience with telehealth and to understand the demographic factors that contribute to telehealth usage.

## Methods

### The Reimagining Health Care Survey

We constructed a 48-question survey divided into 5 sections corresponding to themes of interest in the research. The 5 sections measured experiences with health care consultations including telehealth; willingness or ability to use technology to support one’s own health; willingness to share one’s own information on health and conditions to support one’s own health; willingness to share one’s own information on health and conditions to support others; and willingness to adopt alternative methods of care. The survey questions were based on a modification of the Deloitte US Health Care Consumers survey [14].

This paper focuses on the first section, experiences with health care consultations including telehealth. The section includes 7 questions, with questions 1 to 4 focusing on the frequency of health or telehealth consultations and types of clinical consultations, including general practitioners (GPs), specialists, allied health professionals, or nurses; question 5 focusing on experience with telehealth; question 6 focusing on the quality of telehealth consultations and technology; and question 7 focusing on satisfaction with telehealth. Questions 1 to 4 used direct responses relevant to the question. Questions 5 to 7 used a Likert scale to collect responses (strongly disagree, disagree, neither agree nor disagree, agree, strongly agree). The remaining 4 sections addressed broader aspects of digital health; published findings from these sections can be found in our previous publication, by Lee and colleagues [13].

### Study Population

We conducted a national survey between the period of June 5, 2021, and September 13, 2021. The survey was distributed to individuals aged 18 years or older residing in Australia through the networks of the collaborating project partners including the Consumer Health Forum (CHF), Deloitte Australia, the Digital Health Cooperative Research Centre (DHCRC), and Curtin University. The partners distributed a web link to participate through subscribed distribution lists and social media accounts (Facebook, LinkedIn). The survey included 48 questions related to digital health and was rolled out through the Qualtrics survey portal. To ensure only 1 response was received per participant, we accepted only 1 response per IP address through Qualtrics. In parallel, the survey was distributed to a consumer group through the Australian Health Panel, which includes a subscribed group of consumers available to participate in health-related surveys. For this distribution the survey was divided into 2 separate surveys—survey 1 included questions 1 to 21, and survey 2 included questions 22 to 48. Consumers were invited to complete either survey 1 or 2, or both, but only participants who completed survey 1 or both were included in the final data set. In our interim analysis in August we identified a bias for participants that were female, resided in major cities, or had a bachelor’s degree or higher. At this stage, we conducted an additional rollout through the web-based survey company Dynata to recruit 1000 additional participants that comprised 50% (500/1000) male, 50% (500/1000) female, 64% (640/1000) residing in major cities, 36% (360/1000) residing in regional areas, and 100% (1000/1000) with educational background of below a bachelor’s degree. Participants responding through the Dynata rollout received a one-off US $6.60 payment for participation. All survey participants provided consent to participate, and responses were anonymous. Participants who did not answer (or selected “prefer not to say”) questions on age, area of residence, and level of education were excluded from the study.

### Recruitment Procedures

Invitation to participate from the participating partners was via direct email—from the Consumer Health Forum for the Australian Health Panel, and from Deloitte or the DHCRC to subscribed members (June to September). The partners also publicized the study on social media channels (LinkedIn, Facebook, and Twitter). Additional recruitment took place via survey company Dynata (August to September), who distributed the invitation to subscribed members; participants received US $6.60. Invitations included a brief description of the study and a link to participate on Qualtrics, where the complete participant information sheet was available and consent was collected digitally.

### Statistical Analysis

We reported the percentages of participants who selected a positive response to the Likert scale questions—agree or strongly agree. Logistic regression was used to model the effect of age (66 years or older, with 66 years or younger as the referent) on providing a positive response. Age-adjusted logistic regression was used to estimate the odds ratio (OR) with 95% CI of a positive response associated with educational attainment (up to year 12 graduate, post–high school qualifications, or bachelor’s degree or above) where up to year 12 graduate was the reference group. Age-adjusted logistic regression was repeated for each Australian jurisdiction with at least 100 participants by rurality (major city or inner regional, outer regional, remote, very remote), where the major city was the reference group. All statistical analyses were conducted using Stata or SE (version 14.0 for Windows; Stata Corp).

### Ethics Approval

This study was approved by the Curtin University Human Research Ethics Committee (approval HRE2021-0248). All participants provided informed consent, and data were stored on a secure server with multifactor authentication to access. Participation was anonymous, and no personal identifying information was collected. Participants in the Dynata-recruited cohort received a US $6.60 reimbursement for participation, as per the company procedures.

## Results

### Characteristics of Participants

**Figure 1:**
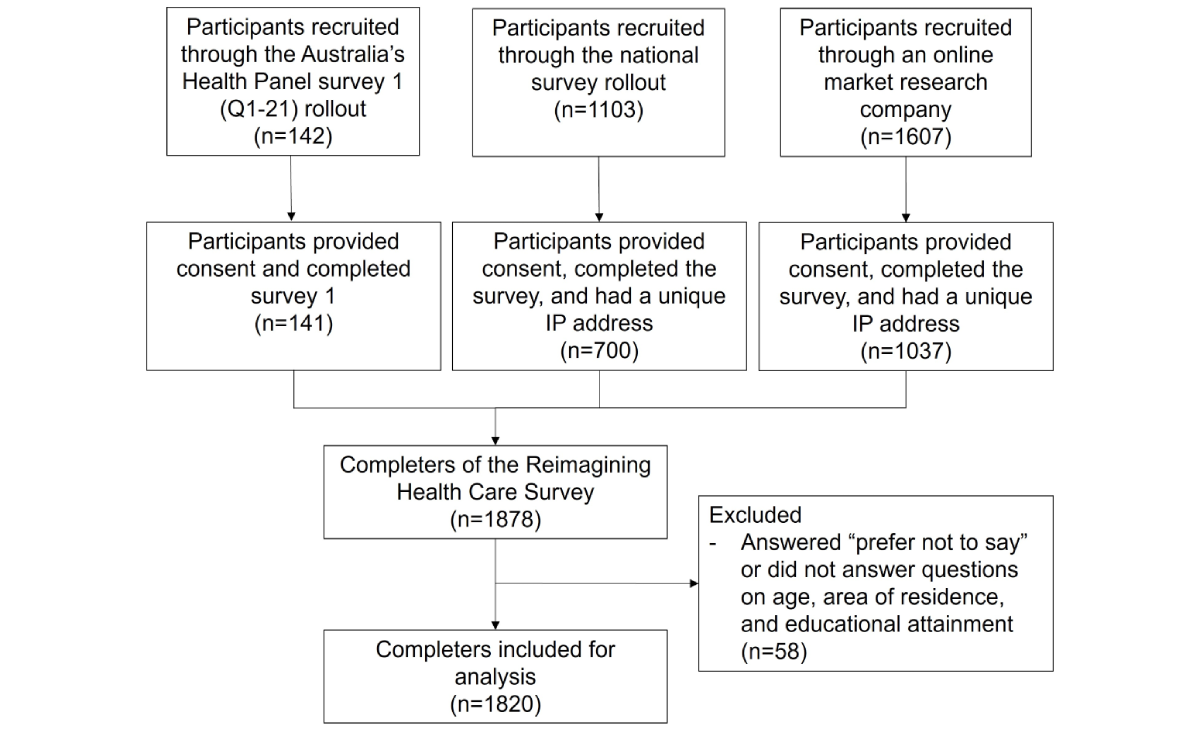
Flow diagram for participant recruitment.

A total of 1820 participants were included in the study analysis after 58 participants were excluded because they did not answer (or answered “prefer not to say”) questions on age, area of residence, or educational attainment (Figure 1). Of these, 37.7% (686/1820) were male and 61.4% (1117/1820) were female, 21.2% (386/1820) were aged 66 years or older, 65.4% (1190/1820) lived in major cities, 9.1% (165/1820) lived in remote or very remote areas, and 33.2% (604/1820) had a bachelor’s degree or above. Full cohort characteristics are summarized in Table 1.

**Table 1:** Characteristics of participants.

		All (n=1820)	Experienced telehealth in past 12 months (n=1114)	Did not experience telehealth in past 12 months (n=493)
**Gender, n (%)**				
	Male	686 (37.7)	362 (32.5)	230 (46.7)
	Female	1117 (61.4)	739 (66.3)	261 (52.9)
	Gender diverse	10 (0.6)	9 (0.8)	1 (0.2)
	Prefer not to say or missing	7 (0.4)	4 (0.4)	1 (0.2)
**Age (years), n (%)**				
	18-25	102 (5.6)	58 (5.2)	28 (5.7)
	26-35	260 (14.3)	163 (14.6)	51 (10.3)
	36-45	332 (18.2)	195 (17.5)	89 (18.1)
	46-55	403 (22.1)	270 (24.2)	91 (18.5)
	56-65	337 (18.5)	194 (17.4)	104 (21.1)
	66-75	240 (13.2)	162 (14.6)	70 (14.2)
	>76	146 (8)	71 (6.4)	60 (12.2)
**State or territory, n (%)**				
	Australian Capital Territory	46 (2.5)	25 (2.2)	18 (3.7)
	New South Wales	620 (34.1)	372 (33.4)	165 (33.5)
	Northern Territory	8 (0.4)	1 (0.1)	5 (1)
	Queensland	294 (16.2)	167 (15)	99 (20.1)
	South Australia	133 (7.3)	78 (7)	44 (8.9)
	Tasmania	40 (2.2)	27 (2.4)	9 (1.8)
	Victoria	472 (25.9)	340 (30.5)	85 (17.2)
	Western Australia	191 (10.5)	92 (8.4)	66 (13.4)
	Other Australian territory	1 (0.1)	0 (0)	0 (0)
	Prefer not to say or missing	15 (0.8)	11 (1)	2 (0.4)
**Remoteness, n (%)**				
	Major city	1190 (65.4)	723 (64.9)	333 (67.6)
	Inner regional	277 (15.2)	170 (15.3)	77 (15.6)
	Outer regional	188 (10.3)	116 (10.4)	41 (8.3)
	Remote or very remote	165 (9.1)	105 (9.4)	42 (8.5)
**Health status, n (%)**				
	Excellent	243 (13.4)	156 (14)	57 (11.6)
	Good	910 (50)	523 (47)	274 (55.6)
	Fair	506 (27.8)	319 (28.6)	125 (25.4)
	Poor	149 (8.2)	111 (10)	33 (6.7)
	Prefer not to say or missing	12 (0.7)	5 (0.4)	4 (0.8)
**Education, n (%)**				
	Up to year 12 graduate	533 (29.3)	283 (25.4)	163 (33.1)
	Trade or technical or vocational training or diploma or associate degree	683 (37.5)	395 (35.5)	193 (39.2)
	Bachelor’s degree or above	604 (33.2)	436 (39.1)	137 (27.8)
**Employment status, n (%)**				
	Permanent full-time	576 (31.7)	358 (32.1)	147 (29.8)
	Permanent part-time	258 (14.2)	146 (13.1)	76 (15.4)
	Contract or temporary	127 (7)	91 (8.2)	25 (5.1)
	Student or studying	60 (3.3)	40 (3.4)	11 (2.2)
	Retired	425 (23.4)	256 (23)	135 (27.4)
	Unemployed	152 (8.4)	70 (6.3)	51 (10.3)
	Unable to work	120 (6.6)	88 (7.9)	26 (5.3)
	Other	78 (4.3)	49 (4.4)	17 (3.5)
	Prefer not to say or missing	24 (1.3)	16 (1.4)	5 (1)
**Annual household income (US $), n (%)**				
	≤16,500	201 (11)	113 (10.1)	55 (11.2)
	16,501-33,200	371 (20.4)	214 (19.2)	106 (21.5)
	33,201-66,500	496 (27.3)	307 (27.6)	130 (26.4)
	66,501-133,000	386 (21.2)	246 (22.1)	99 (20.1)
	>133,000	161 (8.9)	106 (9.5)	44 (8.9)
	Prefer not to say or missing	205 (11.3)	128 (11.5)	59 (12)
Aboriginal or Torres Strait Islander, n (%)		45 (2.5)	30 (2.7)	9 (1.8)
Lesbian, gay, bisexual, transgender, intersex, queer/questioning, asexual, other, n (%)		123 (6.8)	89 (8)	24 (4.9)
Culturally and linguistically diverse, n (%)		83 (4.6)	59 (5.3)	16 (3.3)
Person with a disability, n (%)		240 (13.2)	178 (16)	53 (10.8)

### Frequency of Telehealth Consultations and Distribution of Clinical Types of Consultations (Questions 1-4)

Of the 1820 participants who completed the survey, 88.3% (1607/1820) had engaged in a health care consultation of some type in the previous 12 months. Of those, 69.3% (1114/1607) had engaged in a health care consultation using telehealth. The most common type of consultation was with a GP (959/1114, 86.1%), followed by specialist (387/1114, 34.7%), allied health professional (238/1114, 21.4%), and nurse (64/1114, 5.7%).

Older people (66 years old and above) were more likely to have had a health care consultation (OR 2.54, 95% CI 1.61-4.01) but were less likely to have had that consultation by telehealth (OR 0.74, 95% CI 0.58-0.95). In each Australian jurisdiction where there were at least 100 participants, people outside major cities were no more likely to have had a health consultation in the last 12 months, with the exception of Western Australia, where rural populations were significantly less likely to have had a consultation (age-adjusted OR 0.27, 95% CI 0.12-0.62). However, there was no significant difference between major city or outside-major city populations for the use of telehealth for the most recent health care consultation. Relative to people with up to year 12 education, people with a bachelor’s degree or above were significantly more likely to have accessed telehealth for their most recent health consultation (age-adjusted OR 1.85, 95% CI 1.41-2.43).

Using a Likert scale, we asked questions about the quality of the respondents’ telehealth consultations compared to face-to-face consultations.

### Experience of Telehealth Relative to Face-to-Face Consultations (Question 5)

The majority of participants agreed or strongly agreed that they had received the information they required from their consultations (977/1114, 87.7%). More than two-thirds of participants agreed or strongly agreed that the outcome of their consultations was the same as it would have been face-to-face (797/1114, 71.5%), that the doctor or health care provider made them feel comfortable (931/1114, 83.6%), and that the doctor or health care provider was equally as knowledgeable as providers they have seen in person (924/1114, 82.9%). More than half of participants agreed or strongly agreed that the wait time was shorter than for a face-to-face consultation (753/1114, 67.6%), and that if it was not for the telehealth appointment, they would not have been able to see the health care provider (629/1114, 56.5%). Compared to people with less than a bachelor’s degree, people with a bachelor’s degree or higher were more likely to agree or strongly agree that they had received the information they required from their consultation (age-adjusted OR 1.60, 95% CI 1.02-2.50), and that their doctor or health provider was equally as knowledgeable as other providers they have seen in person (age-adjusted OR 1.56, 95% CI 1.05-2.32).

### Quality of the Telehealth Interaction and Technology (Question 6)

With regard to the quality of the consultations, 67% (746/1114) agreed or strongly agreed that their telehealth consultation was the same quality as face-to-face, but only 20.4% (227/1114) agreed or strongly agreed that their telehealth consultation was higher quality than face-to-face. Less than a fifth of respondents agreed or strongly agreed that they had experienced technical difficulties in accessing their telehealth consultation (194/1114, 17.4%), that the quality of the telehealth consultation affected their interaction (180/1114, 16.2%), or that they felt more nervous using telehealth over a face-to-face consultation (182/1114, 16.3%); 79% (878/1114) of respondents agreed or strongly agreed that they saved a lot of time using telehealth over a face-to-face consultation.

People with a bachelor’s degree or above were less likely to agree or strongly agree that their health care consultation was higher quality (age-adjusted OR 0.47, 95% CI 0.31-0.70), that technical quality of the telehealth consultation affected the meeting (age-adjusted OR 0.61, 95% CI 0.40-0.93), or that they felt more nervous using telehealth versus face-to-face (age-adjusted OR 0.35, 95% CI 0.23-0.54). Conversely, people with a bachelor’s degree or above were more likely to agree or strongly agree that they saved a lot of time using a telehealth appointment versus face-to-face (OR 2.21, 95% CI 1.52-3.22).

### Satisfaction With Telehealth (Question 7)

Just over two-thirds of participants agreed or strongly agreed that they were equally as satisfied with their telehealth consultation relative to face-to-face (765/1114, 68.7%), 60.2% (671/1114) reported that they would like to continue using telehealth to meet their health care needs, and 70.3% (783/1114) of respondents agreed or strongly agreed that they would recommend telehealth to others. There was a bias for preference among people with a bachelor’s degree or above, who were more likely to agree or strongly agree that they would like to continue using telehealth to meet their health care needs (age-adjusted OR 2.03, 95% CI 1.49-2.78), and that they would recommend telehealth to others (age-adjusted OR 2.24, 95% CI 1.60-3.14).

## Discussion

### Principal Findings

In this national survey, we show that over two-thirds of Australians accessing health care had accessed care via telehealth. Of the 1820 participants who completed the survey, 88.3% (1607/1820) had engaged in a health care consultation of some type in the previous 12 months. Of those, 69.3% (1114/1607) had engaged in a health care consultation using telehealth. The most common type of consultation was with a GP, followed by a specialist, allied health professional, and nurse. Older people were less likely to have used telehealth over face-to-face consultation compared to the rest of the population, and there was no difference between metropolitan and nonmetropolitan populations in terms of use of telehealth. People with a bachelor’s degree or higher were more likely to have engaged in telehealth for their health care and to have been satisfied with this mode for their care.

By far the most common type of consultation accessed using telehealth was with GPs, which reflects the typical nature of this type of care, as it may not require the same level of physical examination that specialists or allied health professionals, such as physiotherapists, would require. A GP consultation might typically involve a basic collection of symptoms and concerns from the patient, which may lead to a face-to-face consultation or referral to further care, or simply require a prescription and follow-up where relevant. These activities can reasonably be conducted safely by telehealth; however, work done by Olayiwola and colleagues [15] indicates that appropriate training and guiding principles are required to enable GPs to best serve their patients remotely [15].

We note that the survey period was conducted during the COVID-19 pandemic, when different jurisdictions were facing different levels of restricted access to care and individuals may have had concerns over attending a health care consultation. In this context, individuals may have chosen telehealth to access a GP to discuss concerns over COVID-19 symptoms or to seek a referral for testing in jurisdictions that required it. Similarly, while specialist consultations by telehealth represented less than half of those by GPs, 34.7% (387/1114) of participants reported having had a telehealth consultation in the last 12 months. This seems high relative to the general population and may reflect the shift of consultations to telehealth where possible to avoid unnecessary interaction during the COVID-19 pandemic. The outcomes of patients engaging in telehealth consultations as a result of these changes will be an interesting topic for future investigation, which may lead to permanent changes in the way clinicians engage with their patients [12,16].

It was unsurprising to learn that older people were less likely to have used telehealth, despite being more likely to have accessed health care at all. This likely reflects a more typical disengagement with technology and lack of familiarity with remote communication in general. Since the aging population places a significant burden on the health system, making care accessible to older people is an unavoidable imperative for the future. Indeed, aged care physicians in the United States have noted the benefits of telehealth for aged care beyond the pandemic but remain concerned about equity of access and barriers to engagement [17]. Our results support this need for better support for older people to access remote modes of care. This may be overcome by providing specialized administrative support to assist patients with engagement and dedicated care coordinators who can assist patients to be prepared for their consultations.

It is a common belief that people in regional or remote areas are less engaged in technology and therefore less likely to use telehealth as a mode for seeking health care. Our results show that there was no difference between people in major cities and people outside major cities with regard to their engagement with telehealth. With the exception of Western Australia, people outside of major cities were also not less likely to have engaged in a health care consultation at all. While it is known that people in regional and remote areas experience poorer health outcomes across a wide spectrum of clinical conditions, including chronic diseases and cancer [18], based on our results it does not appear that access or engagement with telehealth is a contributing factor to this. However, given that remote models of care like telehealth are relatively new, it is probably premature to assess their contribution to health outcomes in any population.

The vast majority of respondents reported a positive experience with telehealth with regard to the information they received and their comfort with and confidence in the health professional consulting them. More than half also reported that their wait time was shorter than a face-to-face consultation, and that if it were not for access to telehealth, they would not have been able to see a health care provider. It is a staggering statistic to consider that more than half of the population may be dependent on telehealth to access care, and this is a strong support for the rollout of this mode of care to make it more accessible across the population. However, it is also worth noting that COVID-19 restrictions may have contributed to people’s access to care and choices when faced with the option of face-to-face or remote care.

Relative to face-to-face consultations, a majority of participants felt that their telehealth consultation was equal in terms of the quality of the consultation, and this supports previous research [19]. While technical limitations, such as phone delays or internet speed, may contribute to lower-quality consultations, it does not appear from our results that these are impacting a majority of the population. We also found no relationship between the quality of the consultation or comfort with the technology and regional or remote location, indicating that services are available and adequate in these locations. However, we note that the web-based forum of this survey naturally selects individuals that have access to the internet, and this may represent a bias for people with access to and familiarity with telehealth. We also note that while it was a minority, there remained a significant proportion of individuals reporting a negative response with regard to quality or technology issues with their telehealth consultation. Identifying these populations and modifying care appropriately or providing better services or education is essential if Australia is to adopt telehealth more widely as an equitable means of care. This approach is supported in reviews by Taylor and colleagues [5] and Bailey and colleagues [20], which highlights the need for tailored models of care that engage the patient voice in their design [5,20].

In our opinion, the most poignant finding in this study is the relationship between educational background and the use and experience of telehealth. People with a bachelor’s degree or above were significantly more likely to both access telehealth and report that they would continue using telehealth for their health care needs as well as recommend it to others. People with a bachelor’s degree or above were also more likely to say they saved time by using telehealth, which may be reflective of a population that is more likely to have a busy working life and less flexibility to attend a face-to-face consultation. The preference for positive responses among the more educated population may also be due to a greater likelihood that these people are familiar with technology and are more fluent in remote communication. What is more important in this finding is that there is a clear need for more resources to make telehealth more useable in populations with less than a bachelor’s degree in their educational background. This will represent a majority of the population, highlighting that there remain gaps in telehealth services that may be compromising the quality and accessibility of services to this group. It is known that people from a lower socioeconomic background are at higher risk of conditions such as chronic diseases [21,22] that would benefit from access to regular care, emphasizing the need to improve telehealth services more broadly.

### Limitations

Several study limitations impacted our interpretation of the results. First, the study was conducted during the COVID-19 pandemic, which does not necessarily reflect a period of typical human behavior and definitely does not reflect typical health service availability. Concerns around infection and mandated lockdowns may have impacted peoples’ choice to access telehealth or not, limiting extrapolation of results to the postpandemic environment. Second, we used digital advertising and recruitment for the study so there may have been some bias in the participating population, where people more digitally engaged may be more inclined to engage in a digital form of health care. Finally, the length of the survey was limited to what is reasonable for a person to complete voluntarily. As such the questions focused on areas of primary interest to the research group and may not reflect the full spectrum of investigation that is identified as relevant across the literature. This includes the decision to not differentiate between service modality and grouping telephone and video access into 1 group.

### Conclusions

A majority of respondents in our study cohort had used telehealth as a means of health care in the last 12 months, and the majority provided positive responses with regard to their experience and quality of the consultation. A majority of people were satisfied with their telehealth consultation and would like to use telehealth in the future. There was no relationship between telehealth use, experience or satisfaction, and geographical location; however, older people were less likely to access telehealth services. People with a bachelor’s degree or above were more likely to report a positive experience in all aspects of telehealth, highlighting a need to improve telehealth service access and usability across the broader population. These findings highlight the gaps in telehealth use in the Australian population, and the need for more equitable access to care across the population. The outcomes of the study may be useful in informing future health service design to include additional support to older people and people with a lower educational background.

## Abbreviations

CHF: consumer health forum
DHCRC: Digital Health Cooperative Research Center
GP: general practitioner
OR: odds ratio

## Data Availability

The data sets generated during and/or analyzed during this study are available from the corresponding author on reasonable request.
